# Intergrative metabolomic and transcriptomic analyses reveal the potential regulatory mechanism of unique dihydroxy fatty acid biosynthesis in the seeds of an industrial oilseed crop *Orychophragmus violaceus*

**DOI:** 10.1186/s12864-023-09906-0

**Published:** 2024-01-03

**Authors:** Changfu Jia, Qiang Lai, Yiman Zhu, Jiajun Feng, Xuming Dan, Yulin Zhang, Zhiqin Long, Jiali Wu, Zeng Wang, Xiner Qumu, Rui Wang, Jing Wang

**Affiliations:** Key Laboratory of Bio-Resource and Eco-Environment of Ministry of Education, College of Life Sciences, Sichuan University, Chengdu, China

**Keywords:** *Orychophragmus violaceus*, Dihydroxy fatty acids, Lubricant oil, Seed oil, Multi-omics, Transcription factor, Metabolic regulatory network

## Abstract

**Background:**

*Orychophragmus violaceus* is a potentially important industrial oilseed crop due to the two 24-carbon dihydroxy fatty acids (diOH-FA) that was newly identified from its seed oil via a ‘discontinuous elongation’ process. Although many research efforts have focused on the diOH-FA biosynthesis mechanism and identified the potential co-expressed diacylglycerol acyltranferase (*DGAT*) gene associated with triacylglycerol (TAG)-polyestolides biosynthesis, the dynamics of metabolic changes during seed development of *O. violaceus* as well as its associated regulatory network changes are poorly understood.

**Results:**

In this study, by combining metabolome and transcriptome analysis, we identified that 1,003 metabolites and 22,479 genes were active across four stages of seed development, which were further divided into three main clusters based on the patterns of metabolite accumulation and/or gene expression. Among which, cluster2 was mostly related to diOH-FA biosynthesis pathway. We thus further constructed transcription factor (TF)-structural genes regulatory map for the genes associated with the flavonoids, fatty acids and diOH-FA biosynthesis pathway in this cluster. In particular, several TF families such as bHLH, B3, HD-ZIP, MYB were found to potentially regulate the metabolism associated with the diOH-FA pathway. Among which, multiple candidate TFs with promising potential for increasing the diOH-FA content were identified, and we further traced the evolutionary history of these key genes among species of Brassicaceae.

**Conclusion:**

Taken together, our study provides new insight into the gene resources and potential relevant regulatory mechanisms of diOH-FA biosynthesis uniquely in seeds of *O. violaceus,* which will help to promote the downstream breeding efforts of this potential oilseed crop and advance the bio-lubricant industry.

**Supplementary Information:**

The online version contains supplementary material available at 10.1186/s12864-023-09906-0.

## Background

Modern crop cultivated populations have been shown to only contain about 6% of the genetic diversity compared with those found in the gene pool of wild species [1]. In addition, wild species contributed to the majority of our currently cultivated plants via long-term domestication within the past 12,000 years [2]. Owing to the rich genetic variation contained in wild species, they showed great potential for expanding the design space for future crop varieties, especially in the pace of rapid climate change [3–5]. As sessile organisms, plants from specific species or lineage can produce different metabolites derived from divergent compounds or pathways to facilitate them adapting to local abiotic and biotic challenges, and on the other hand, it also provides valuable metabolite resources for industrial, medical and agricultural interests [6–8]. For example, whole genome duplication event (WGD) provides the opportunity for Opium poppy to produce multiple gene copies to ultimately increase the production levels of lineage-specific substrate, morphine, which is widely used in medicine through a punctuated patchwork model [9, 10]. In addition, numerous genes related to camptothecin biosynthesis in *Camptotheca acuminata*, which is benefit for treating malignant tumors, are derived due to the lineage-specific WGD [11]. Recently, a novel pathway for synthesizing two C24 di-hydroxy fatty acids (nebraskanic fatty acid, 7,18-OH-24:1Δ^15^; wuhanic fatty acid, 7,18-OH-24:2Δ^15,21^) was identified from *Orychophragmus violac*eus [12]. These fatty acids make the seed oil of *O. violaceus* more stable in high-temperature than castor oil, a widely used plant-based lubricant resource [12, 13], highlighting its industrial properties. *O. violaceus*, also known as ‘er-yue-lan’ in China [14], is an annual or biennial shade-tolerance plant in the family of Brassicaceae [15]. It has wide natural distribution areas, ranging from southwest to northeast of China, and extending into Korea. [16, 17]. This plant typically features clustered small purple flowers although some individuals contain white or yellow flower color, and it has been widely used for urban afforestation in many cities as well [18]. Multiple field experiments showed that *O. violaceus,* when intercropped with some other main crops, have the potential to diminish the need for nitrogen application in the soil while simultaneously enhancing overall crop productivity [19–21]. As an evolutionarily close species relative to *Brassica* [22, 23], *O. violaceus* has long been served as potential oil crops because of its high oil contents [24, 25] and was widely used as backcrossing progenies for improving the genetic sources of *Brassica* crops owing to its high seed yield potential and desirable oil quality [26, 27]. Therefore, the wild species *O. violaceus* has great potential to be further developed as an oil species for planting or intercropping with other main crop species. In particular, understanding of the genetic mechanism associated with the biosynthesis pathways of the two unique very-long-chain hydroxy fatty acids (diOH-FA) is urgently needed.

The quality of *O. violaceus* seed oil was highly dependent on the content of several metabolic pathways influencing the DiOH-FA content. Firstly, as two recent genome assemblies of *O. violaceus* [28, 29] showed that it has undergone a unique WGD, the neofunctionalization of one copy of *FAD2* of *O. violaceus* together with the two WGD copies of *FAE1* likely cause the born of diOH-FA directly [12]. Secondly, fatty acid biosynthesis provides the upstream substrate oleoyl-phosphatidylcholine (PC) that was used as upstream precursor for the diOH-FA biosynthesis and is positively correlated with seed oil content (SOC). Thirdly, owing to the shared common precursor (malonyl-CoA) between fatty acid and flavonoid synthesis pathways [30], the diOH-FA content could be also indirectly influenced by genes related to the flavonoid biosynthesis. In contrast to fatty acid synthesis, flavonoid synthesis produces proanthocyanidins (PAs) that was mainly associated with the seed coat content (SOG) but negatively correlated to SOC [31, 32]. Multiple studies showed that when knocking out the key genes of flavonoid synthesis such as *TT2*, *TT4*, *TT8*, the seed color could change from black to yellow in *B. napus*, and the fatty acid content was significantly increased in the seed [33–35]. Finally, the storage of diOH-FA involving in triacylglycerol (TAG) biosynthesis also plays an important role in improving the seed oil quality for superior lubrication properties [13, 29].

Numerous domesticated crops such as maize, cotton, common bean and tomato, have undergone significant transcriptional reprogramming, especially given the fact that many of the domestication genes are transcription factors [36–40]. As a result, in order to improve the seed oil quality of *O. violaceus* for its industrial properties, it is crucial to investigate the transcriptional regulatory networks that likely play a key role in controlling the expression patterns of the structural genes involved in diOH-FA. Integrative analysis of multi-omics data including metabolome and transcriptome were successfully used in identifying gene functions and characterizing metabolic pathways in plants [41, 42]. Through the integrative analysis of metabolic and regulatory networks, multiple studies have identified key transcription factors that regulate the desired traits in agriculture and horticultural crop species [43–46]. For example, a MicroTom tomato metabolic regulatory network (MMN) constructed by the combination of metabolome and transcriptome identified two novel transcription factors that regulated the steroidal glycoalkaloid and flavonoid metabolism [44]. Another study constructed a kiwifruit metabolic regulatory network (KMRN) and found links between the landscape of metabolic changes through 11 fruit developmental and ripening stages [43]. These studies provide high-effective ways for improving the quality of key trait of crop species. Recently, two high-quality reference genomes of *O. violaceus* have been published and this provide opportunity to apply multi-omics technology to dissect the genetic basis of seed development of *O. violaceus* [28, 29]. Furthermore, compared with other sequenced species in Brassicaceae, *O. violaceus* has a relatively large genome around 1.3Gb and multiple evidences showed that *O. violaceus* have undergone a lineage-specific WGD event [28, 47]. New compounds were usually born through WGD, such as the production of camptothecin and morphinan could all be attributed to their lineage-specific WGD [9, 11]. Previous studies found that the new born of specific di-OH FA in *O. violaceus* could also be ascribed to the neofunctionalization of a copy of *FAD2* genes and two WGD copies of *FAE1* which are essential for producing di-OH FA [12]. However, these studies only focused on several structural genes directly involved in di-OH biosynthesis and further investigation is highly needed for all the pathways associated with di-OH fatty acids as we mentioned above.

In this study, we utilized and integrated transcriptomes and metabolomes datasets during four different stages of seed development based on our newly assembled reference genome [48]. In total, we detected 1,003 metabolites and 22,479 expressed genes among four different seed developmental stages of *O. violaceus*. To reach a more accurate gene functional annotation data set, we used a systematical gene identification approach to draw specific evolutionary history for each orthogroup in the family Brassicaceae [49]. We constructed gene regulatory networks of lubrication oil related pathways and detected the gene expression and metabolic dynamic processes during seed developmental stages. Finally, we identified key TFs potentially regulate the di-OH FA content.

## Material and methods

### Sample collection

The *O. violaceus* plants were cultivated at the Wangjiang campus of Sichuan University, Chengdu, China. We marked the appearance of the first flower as the start point, and days after flowering (DAFs) were used as time points. We collected seeds in the siliques of four different developmental stages (21–63 DAF) at around 4:00 pm and then extracted the seeds of the siliques to liquid nitrogen for further transcriptome and metabolome sequencing with four biological replicates. The mature tissues including flower, stem, root and leaf were also collected with three biological replicates for further RNA sequencing.

### RNA sequencing and analysis

RNA sequencing of seeds in four different developmental stages with each having four biological replicates and mature tissues with each having three biological replicates was performed by the Beijing Genomics Institute (Shenzhen, China). Around 0.5g of each sample was used to extract RNA for transcriptomics sequencing. For data analysis, clean reads of all samples were trimmed using fastp with default parameters [50]. Trimmed paired reads were mapped to *O. violaceus* genome using HISAT2 software [51]. HT-SEQ [52] was used for counting mapped reads of each gene with ‘–mode = union –nonunique = none -s no –secondary-alignments = ignore’ parameters, and then transcripts per million (TPM) were calculated via home-based R script.

### Metabolome profiling

To obtain the metabolite extracts of seeds in four different developmental stages, the seeds samples were collected, freeze-dried, crushed, weighed, dissolved, centrifuged, absorbed, and filtrated. Then, the extracts were analyzed using an UPLC-ESI–MS/MS system (UPLC, SHIMADZU NexeraX2, www.shimadzu.com.cn/; MS, Applied Biosystems 4500 Q TRAP, www.appliedbiosystems.com.cn/). We used Analyst v1.6.3 software to perform the qualitative and quantitative analyses for raw data produced via UPLC-MS/MS and the details of the whole schedule followed the multiple reaction monitoring method [53].

### Co-expression/co-regulation cluster identification and regulatory network construction

Co-expression/co-regulation analysis was performed on different seed developmental stages based on K-means method [54] using ClusterGVis package (https://github.com/junjunlab/ClusterGVis). Principal component analysis (PCA), hierarchical clustering analysis (HCA) and heatmaps were performed using PRCOMP, hclust and pheatmap function in R(www.r-project.org/), respectively. We defined gene promoter region of structural genes as 2000bp upstream to the start of the transcription start site, and then predict transcription factor binding sites (TFBS) in the promoter regions and transcription factors (TFs) in *O. violaceus* genome using plantTFDB website [55]. The TF-related gene regulatory networks were generated by combining Pearson correlation coefficient (PCC > 0.95, *p *value < 0.05) between transcription factors and structural genes and also the availability of TFBS present in the promoter regions of structural genes in the same cluster. The TF-gene regulatory networks were visualized by CYTOSCAPE [56]. Kyoto Encyclopedia of Genes and Genomes (KEGG) [57–59] analysis were conducted by clusterprofiler4 software [60].

### Identification of structural genes of diOH-FA biosynthesis related pathway and phylogenetic analysis

Given the relatively close phylogenetic relationship and robust genome collinearity between *A. thaliana* and *O. violaceus*, and also the availability of numbers of high-quality genomes from species in the family Brassicaceae, we followed a powerful method as performed in salmonids species [49] for ortholog inference in order to identify the crucial biosynthesis genes we mainly focused. In brief, combining our gene annotation files of *O. violaceus*, we downloaded gene annotation files of other 14 Brassicaceae species, and then extracted the single longest protein as representation of the corresponding gene. All the single longest proteins of each species were used in OrthoFinder analysis [61] to assign gene ortholog groups (orthogroups). We aligned the corresponding CDS sequences in each orthogroups using MACSE software [62] and then generated and realigned against the species tree using TreeBest (https://github.com/Ensembl/treebest). Gene trees in each orthogroup were split at the level of monophyletic Brassicaceae clades, and were then filtered by their tree topologies. Based on the filtered orthogroups information, we used structural genes of diOH-FA biosynthesis related pathways of *A. thaliana* to query corresponding homologous genes in *O. violaceus*. For the phylogenetic analysis of a specific gene, such as *FAE1* gene, we located its orthogroups and then extracted gene trees constructed by previous method and visualized in R. The WGD genes were identified based on the collinearity between duplicated blocks by WGDI software [63].

### Gene family identification and associated evolutionary analysis

We downloaded the multi-alignment files of FAE1_-CUT1-RppA domain [PF08392] from Pfam database [64] (http://pfam.xfam.org/) and performed HMM search against query genomes using HMMER v3.0 software [65]. The *KCS* family members of *A. thaliana* were also downloaded from TAIR10 and BLASTp [66] using evalue < 1e-5 as cutoff against our query genomes. The intersection of these two methods mentioned above were used for downstream analysis. The multi-alignment for genes matrix were done by MAFFT7 program [67], and IQ-tree2 [68] was used for constructing phylogenetic tree.

## Results

### Construction of* O. violaceus* metabolic regulatory network

**Fig. 1:**
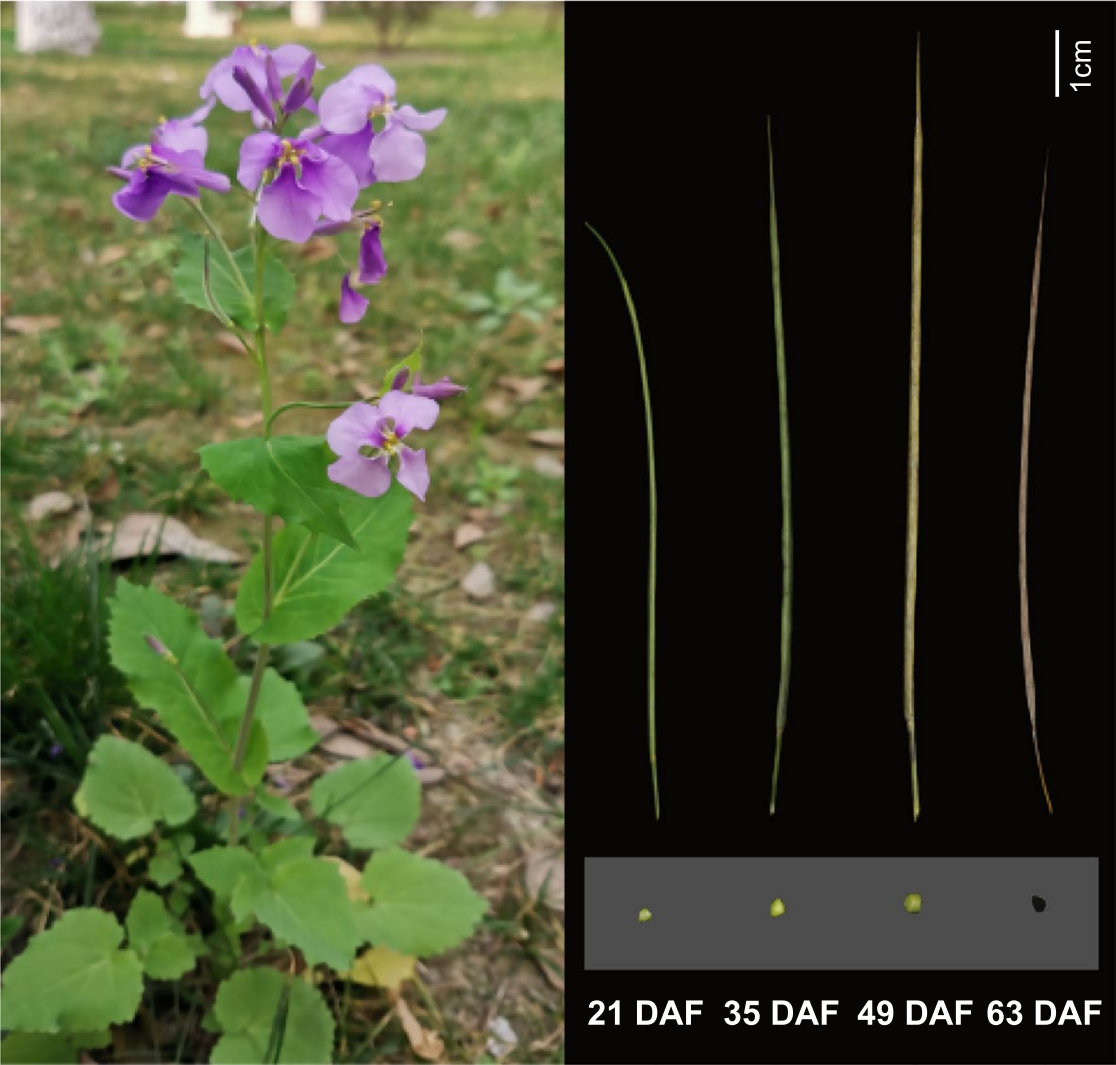
Whole seedling of *O. violaceus* and the sampling strategy for performing integrative metabolomic and transcriptomic analyses during four stages of seed development ranging from 21 days after flowering (DAF) to 63 DAF in this study

To investigate the genetic mechanism influencing diOH-FA content of *O. violaceus*, we parallelly conducted transcriptome and metabolome analysis for the four seed development stages of *O. violaceus*, ranging from days after flowering (22 DAF), 35 DAF, 47 DAF and 63 DAF with each growth time having four biological replicates (Fig. 1). In addition, four mature tissues including leaf, root, flower, stem with three biological replicates were added to perform RNA-sequencing to improve the resolution for detecting relationships between transcription factors and structural genes related with seed oil content and quality.

**Fig. 2:**
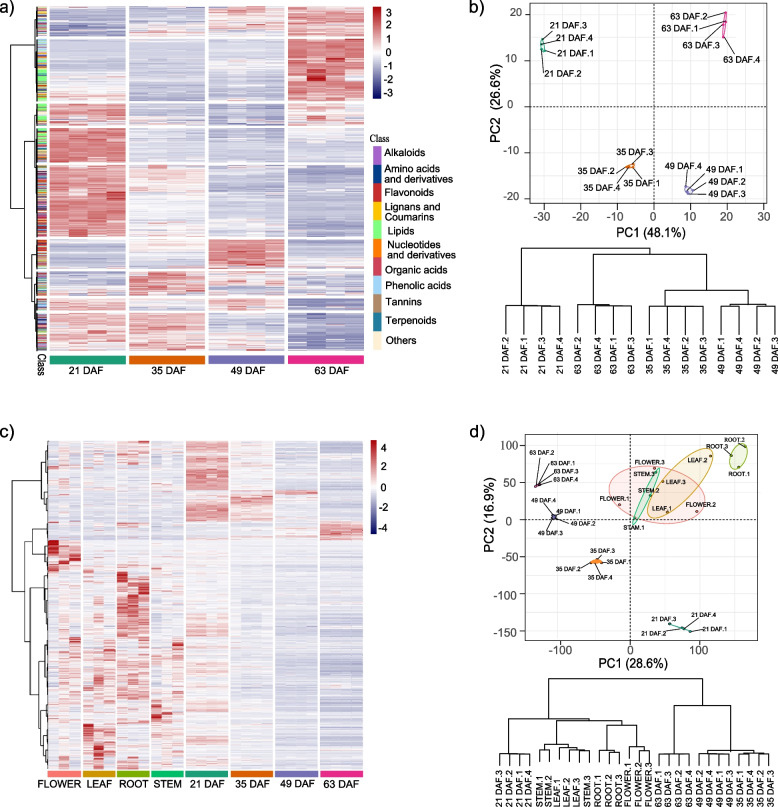
Summary of metabolome and transcriptome data in *O. violaceus*. (**a**) Overview clustering of the metabolome data from four different seed developing stages ranging from 21 days after flowering (DAF) to 63 DAF with each having four replicates, and (**b**) principal component analysis (PCA) and dendrogram cluster for these samples. (**c**) The clustering of the transcriptome data from flower, leaf, root, stem with each having three biological replicates together with the four different seed developing stages with each having four biological replicates, and (**d**) the corresponding PCA and dendrogram cluster analysis based on the transcriptome dataset

A total of 1,003 distinct annotated metabolites were identified in *O. violaceus* seeds, including 161 phenolic acids, 154 lipids, 140 flavonoids, 85 amino acids and derivatives, 75 alkaloids, 75 organic acids, 55 nucleotides and derivatives, 43 terpenoids, 39 lignans and coumarins, 7 tannins and 169 additional compounds that could not be classified into above 10 main classes (Table S1, Fig. 2a). Analysis of these 1,003 metabolites among different seed developmental stages showed that lipids accumulate preferentially in early stage (22 DAF) and ripening stage (63 DAF) (Fig. 2a). Principal component analysis (PCA) indicated that replicates based on metabolites accumulation pattern could well-divided into four different developmental stages by the first two principal component axes that account for 74.7% of total variation. In line with the PCA, the cluster dendrogram also showed that samples could be classified into four subgroups according to the four seed developmental stages (Fig. 2b).

For the transcriptome analysis, we sequenced 28 samples and produced a total of 196.65 Gb with 7.02 Gb clean data per sample (Table S2). We mapped each sample to newly assembled genome of *O. violaceus* and then extracted uniquely mapped reads to calculate Transcripts Per Million (TPM) per gene. Nearly half of genes (22,479/49904) of *O. violaceus* genome were found to be expressed in seed (TPM > 3 in at least one sample). Analysis of heatmap, PCA and cluster dendrogram of the transcriptome data all supported the distinct separation across different tissues and seed developmental stages (Fig. 2c, Fig. 2d). Overall, both the metabolome and transcriptome results showed that samples across different seed developing times of *O. violaceus* exhibited distinct metabolite accumulation and gene expression patterns.

### *O. *violaceus metabolome and transcriptome are co-regulated in three main clusters during seed developmental stages

**Fig. 3:**
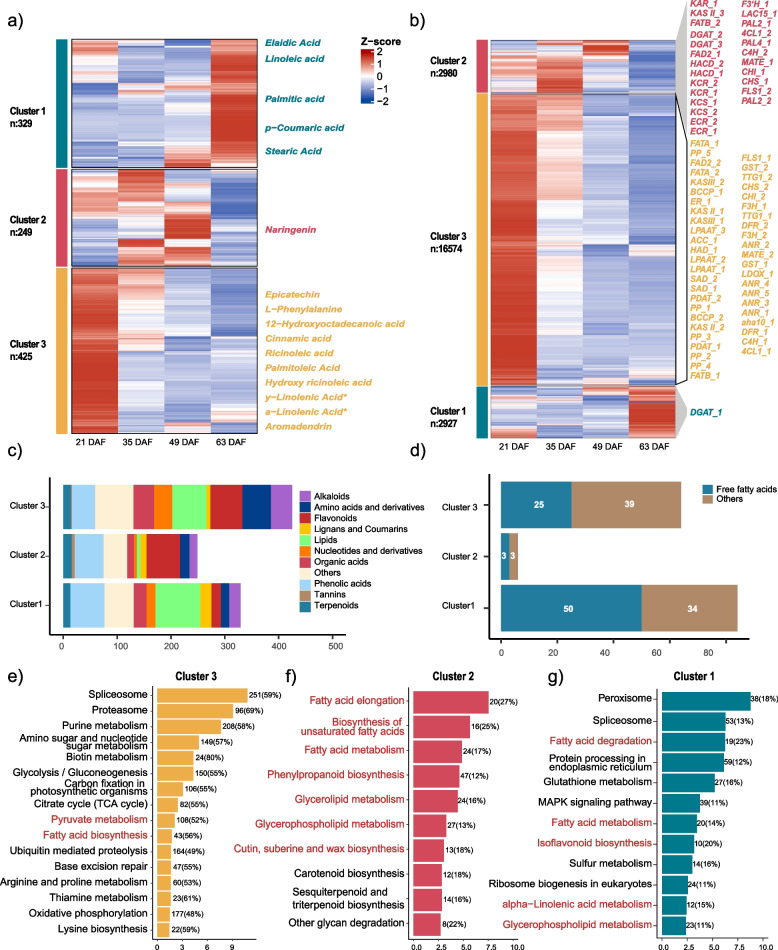
Dynamics of metabolite and gene expression during four different seed developing stages. K-means algorithm grouped the 1103 metabolites (**a**) and 22,479 co-expressed genes (**b**) into three main clusters. Z-score data were standardized to -4 to 4. Statistics of the class of all metabolites (**c**) and free fatty acids (**d**) in 3 clusters. Kyoto Encyclopedia of Genes and Genomes (KEGG) analysis of co-expressed genes in cluster 3 (**e**), cluster 2 (**f**) and cluster 1 (**g**) are shown separately

To unveil the dynamics and relevant genetic mechanisms of the metabolite accumulation at different developmental stages of *O. violaceus* seeds, we classified all 1,003 annotated metabolites and 22,479 expressed genes of seeds into three main clusters based on their accumulation and expression pattern using *K*-means algorithm (Fig. S1, Table S3). These clusters constructed by metabolites and genes expression showed highly consistent pattern, mainly enriched in a specific developing time, such as T1(cluster 3), T4(cluster1), T2 and T3(cluster2) (Fig. 3a, b, Fig. S1). We noticed that different types of compounds preferred to accumulate in different clusters (Fig. 3c). For example, lipids including free fatty acids such as stearic acid and linoleic acid that mainly determine the seed oil quality are highly enriched in cluster 1 (Fig. 3a, c, d), indicating that the seed oil content-related substrates mostly undergone biosynthesis process in the younger stage and then stored in the mature seeds. In contrast to the accumulation pattern of important free fatty acids, flavonoids such as naringenin, epicatechin, phenylalanine which indirectly change the seed oil content, on the other hand, are largely enriched in cluster 2 and cluster 3 (Fig. 3a, c).

Compared to dynamic metabolomes during seed development, we found that most genes were actively expressed in the early stage of seed, mainly in cluster 3. For example, most genes of fatty acid biosynthesis pathway were observed to be enriched in cluster 3, again supporting the intense biosynthesis process in the early seed developing stage as reported also in other studies [69]. Most interestingly, in contrast to genome-wide expression pattern, we observed that all the genes directly involved in diOH-FA biosynthesis such as *FAD2, FAE1, HACD, ECR* were specifically enriched in cluster 2, mainly expressed in the mid-stage of seed development. Meanwhile, diacylglycerol acyltranferases (*DGATs*) that were assumed to be crucial for the storage of diOH-FA were also presented in cluster 2. In line with previous study, these results showed that genes specific to cluster 2 might have an important role on *O. violaceus* seeds oil biosynthesis and storage [29]. In addition, we identified a third copy of *DGAT* located in the cluster 1 (Fig. 3b), which had been overlooked in a previous study [29]. These results suggest an expression divergence among the three *DGAT* copies during different seed development stages. Afterwards, we further performed KEGG enrichment analysis on cluster 3, 2 and 1. The enriched metabolic pathway in cluster 3 include fatty acid biosynthesis and pyruvate metabolism, while the enriched metabolic pathway in cluster 2 include fatty acid elongation, biosynthesis of unsaturated fatty acids, fatty acid metabolism, glycerolipid metabolism and glycerophospholipid metabolism (Fig. 3e, f). Cluster 1 is enriched in fatty acid degeneration, fatty acid metabolism, isoflavoniod biosynthesis, alpha-linolenic acid metabolism and glycerophospholipid metabolism (Fig. 3g). These results indicate that the fatty acid and pyruvate resource were actively synthesis in the early stage and then these substrates further acted as precursor for the diOH-FA biosynthesis and stored in the mid-developing stage. In the late stage of seed development, fatty acid might be degenerated for supply energy and downstream biosynthesis processes were instead active.

### Identification of genes and pathway related to the diOH-FA biosynthesis

**Fig. 4:**
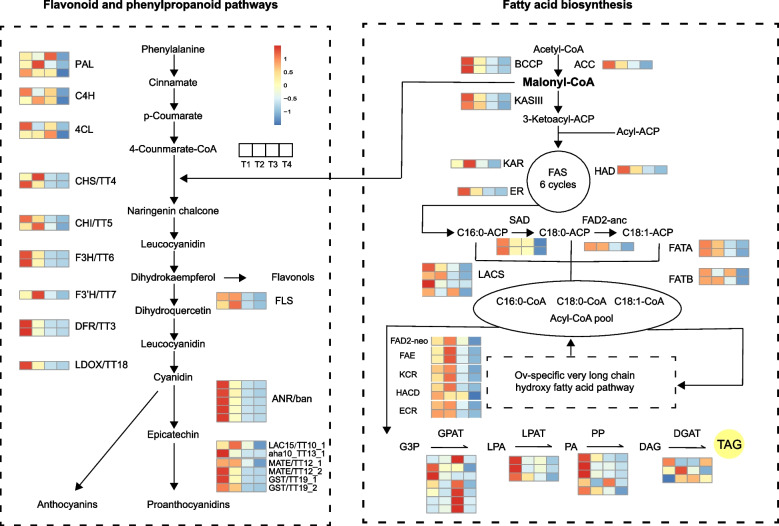
Schematic representation of the unique dihydroxy fatty acids (di-OH FA) biosynthesis related pathway and corresponding structural genes in *O. violaceus*. Expression data along the four different stages of seed development for each gene were standardized to -2 to 2

To further understand the evolutionary and molecular regulatory mechanism associated with the unique diOH-FA biosynthesis in seed oil of *O. violaceus*, we systematically identified the structural genes contributed to the biosynthesis and storage of diOH-FA. Competition between flavonoids biosynthesis and fatty acid biosynthesis for the shared common precursor, malonyl-CoA, determine the negative correlation between flavonoids accumulation and seed oil content. We identified *PAL, C4H, 4CL, CHS/TT4, CHI/TT5, F3H/TT6, F3’H/TT7, DFR/TT3, FLS, LDOX/TT18, ANR/ban, LACS15/TT10, AHA/TT13, MATE/TT12, GST/TT19* genes of flavonoid biosynthesis pathway, *BCCP, ACC, KASIII, KAR, ER, HAD, LACS, SAD, FAD2, FATA, FATB* genes involved in fatty acid biosynthesis pathway, *GPAT, LPAT, PP, DGAT* genes involved in TAG biosynthesis and *FAE1, FAD2, KCR, HACD, ECR* genes involved in *O. violaceus*-specific diOH-FA biosynthesis (Fig. 4, Table S4). At the early stage of seed developing time, the majority of structural genes in fatty acids and flavonoids biosynthesis pathway were actively expressed in T1 or T2 stages and might accumulated the resource precursor for diOH-FA biosynthesis. All the structural genes of diOH-FA, including *FAE1, FAD2, KCR, HACD, ECR* and also one copy of *DGAT* gene shared the same expression pattern that were mainly enriched in T2 stage, indicating the intense diOH-FA biosynthesis process occurred in the mid-stages of seed development. Given the recent history of lineage-specific WGD, we found that most structural genes in the di-OH FA biosynthesis related pathway contained two copies. Similarly, the genes of *F3H, DFR, BCCP, SAD, FATA, FAE* and *HACD* all showed similar expression pattern with higher expression at the early developing stage based on the heatmap plot, implying their conserved regulatory function during seed development (Fig. 4).

### Regulatory networks of diOH-FA biosynthesis-related pathways

**Fig. 5:**
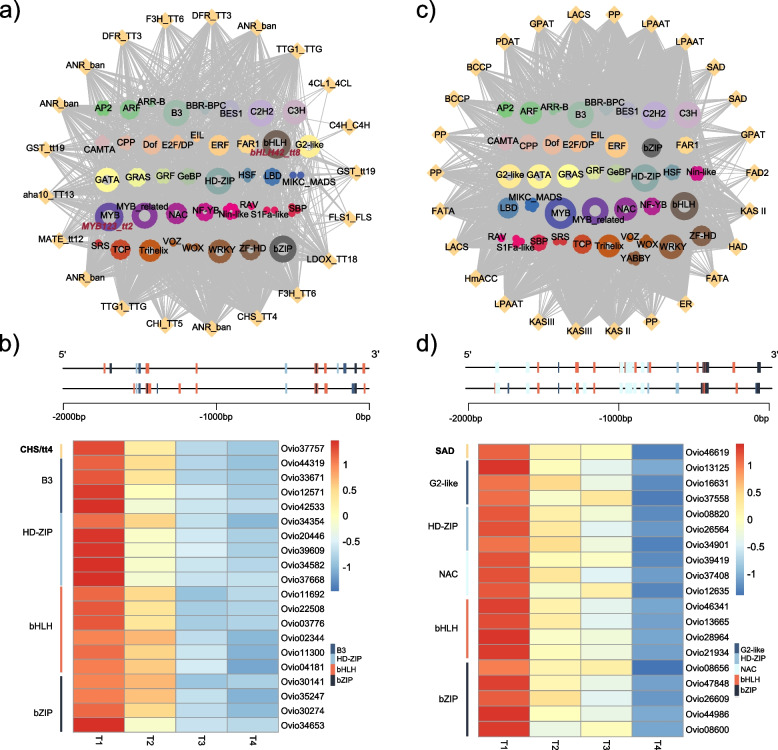
Regulatory networks of flavonoid (**a**) and fatty acid (**c**) biosynthesis pathway constructed by genes in cluster 3. Two structural genes inolved in flavonoids biosynthesis (**b**, *CHS/tt4*) and oleic acid production (**d**, *SAD*) were selected to show the regulatory relationship of them with the selected 19 TF genes that were identified as potential direct upstream regulators. Heatmap representation of average transcripts per million (TPM) values at 4 stages of seed development (T1 to T4). All the expression values were standardized from -2 to 2

As the regulation of flavonoids and fatty acids biosynthesis in the early stage could influence the content of precursor of diOH-FA, the identification of transcription factors (TF) that may regulate the transcriptional regulation patterns of structural genes (SG) play a key role in guiding future manipulation of the key compounds content or desirable crop trait [43–46]. To further study the regulatory process during the early stage of seed development, we extracted the structural genes of flavonoids and fatty acid biosynthesis specifically located in cluster 3 as shown in Fig. 3. Then we calculated the *pearson* correlation coefficient (PCC) using *p *value < 0.05 as cutoff between transcription factors in cluster 3 and these structural genes to construct the TF-SG regulatory networks. At the TF-SG regulatory network of flavonoids biosynthesis, we found bHLH family contained the most members, followed by the GATA and bZIP families (Fig. 5a, Table S5). Among them, consistent with our results shown here, *TT2* gene named *MYB123* from MYB family and *TT8* from bHLH family were previously proved for positively regulating the flavonoids and negatively regulating fatty acid content through knockdown analysis in *B. napus* respectively [34, 35]. This further implies the reliability of our regulatory network constructed for *O. violaceus* (Fig. 5a).

For fatty acid biosynthesis, we found that bHLH family also contained the most members regulating the structural genes, followed by the MYB and B3 families (Fig. 5c). To further investigate the regulatory relationship of the potential transcription factors and its associated structural genes, two genes that could potentially increase the free fatty acid content for synthesize diOH-FA were selected. One is *CHS/TT4*, which was proved to negatively regulate the flavonoids content and positively increase the fatty acid contents [33]. The other one is *SAD*, which catalyzed the first desaturation step leading to oleic acid that is the upstream substrate of diOH-FA [12, 70]. We found that 24 bHLH, 18 HD-ZIP, 16 bZIP and 16 B3 transcription factors showed significantly high correlation with *CHS/tt4*, and we selected a part of them for visualization as shown in heatmap based on their PCC values (Fig. 5b, Table S6). Meanwhile, five members from bZIP, four members from bHLH, three members from NAC, HD-ZIP, G2-like TF families were identified as potentially regulate *SAD* gene expression based on the correlation analysis (Fig. 5d). Apart from the genes mentioned above, we filtered out all the candidate TF genes that might regulate the precursor contents of diOH-FA (Table S5, S6).

### Insight into the spatio-temporal regulation of the unique diOH-FA biosynthetic pathway in *O. violaceus*

**Fig. 6:**
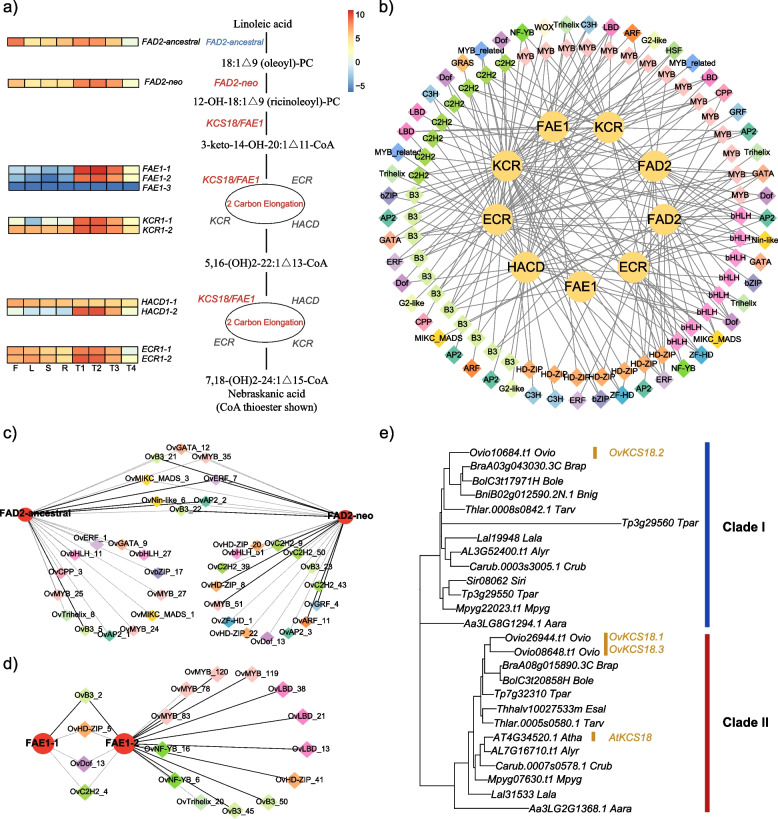
Metabolic pathway for dihydroxy fatty acids (di-OH FA) biosynthesis (**a**) and the associated transcriptional regulatory network (**b**). Sub-network for *FAD2* (**c**) and *FAE1* (**d**) which is crucial for di-OH FA biosynthesis. Circles represented structural genes involved in di-OH FA biosynthesis and diamond with various colors represent different families of transcription factors. (**e**) The evolutionary history of *FAE1* genes in the family Brassicaceae

Given that diOH-FA content determines the seed oil quality of *O. violaceus* for industrious use, we here identified all the structural genes involved in diOH-FA biosynthesis, including *FAD2, FAE1, KCR, HACD* and *ECR*. Except for the *FAE1* genes that have three copies, all the other structural genes involved in diOH-FA synthetic contained two copies, and all of them were produced by the unique WGD of *O. violaceus* through aligning these gene copies to the previous research [29] (Fig. 6a). To further locate the key genes that might potentially regulate the diOH-FA biosynthesis uniquely in *O. violaceus*, we constructed a comprehensive TF-SG gene regulatory network to identify key transcription factors that show great impact on diOH-FA biosynthetic pathways based on multi-tissue mRNA-seq data. Defining PCC > 0.8 & *p* value < 0.05 as the cut-off of the correlation between TF and SG, we found 102 TFs whose expression patterns in selected 8 tissues were highly correlated to the 10 structural genes (Fig. 6b, Table S8). Among the 102 TFs in the gene regulatory network, 37 TFs correspond to MYB, 25 TFs correspondingto B3 and 21 TFs correspondingto C2H2 transcription factors, implying the potential important role of these families in regulating diOH-FA biosynthesis.

As *FAD2* and *FAE1* are the key elements for diOH-FA biosynthesis, we further extracted sub-regulatory networks of *FAD2* and *FAE1* genes. The two *FAD2* copies with different function owing to a crucial amino acid mutation competitively use the same substrate, and accordingly, the *FAD2*-neofunction copy plays a key role during the born of diOH-FA [12]. In the *FAD2* sub-network, we found that some TFs showed highly correlation for both copies of *FAD2*. However, multiple TFs, such as C2H2 members, might specially regulate the neo-function copy of *FAD2* (Fig. 6c), and these novel TFs we identified here needs future deep exploration to decipher their functional roles in regulating the diOH-FA biosynthesis. In addition, *FAE1* gene belongs to *KCS* gene family which elongate the fatty acids to very long chain FA [71, 72]. To trace the evolutionary history of the three copies of *KCS18/FAE1* genes, we identified the *KCS* gene family of *O. violaceus* and used the same strategy also on *A. thaliana* and *B. rapa *(Fig. S2)*.* Phylogenetic analysis showed that *O. violaceus* have retained at least one copy for each of the *KCS* genes to its *A. thaliana* counterpart. More strikingly, although oilseed rape *B. rapa* has undergone a whole genome triplication event, we found that almost all genes in *KCS* family of *O. violaceu* have higher copy numbers than *B. rapa*, indicating that the increased amounts of *KCS* genes might strengthen the power of producing very long chain fatty acids in *O. violaceus*. (Fig. S2, Table S7).

To further trace the evolutionary history of *FAE1/KCS18* gene and explore its potential role in elongating the fatty acids to very long chain FA [71, 72], we extracted the orthogroup of *KCS18* gene across the family Brassicaceae and found that it could be divided into two groups, clade I and clade II (Fig. 6e, Fig. S2, Table S8). *A. thaliana* has lost the copy of clade I while other species remained. The remaining *KCS18* gene of *O. violaceus* from clade I did not express in any tissues we sequenced, and the other two copies of *KCS18* from clade II mainly expressed during seed development stages in *O. violaceus*. In the two expressed *FAE1* sub-network, we found a WGD paired TFs from B3 family named *FUS3* (Ov_B3_50 and Ov_B3_45), which have been certified to impact seed oil content in many other oilseed crops [73, 74]. Both copies of *FUS3* have high correlation with the expression of *FAE1-2* and therefore may be highly related to the regulation of the diOH-FA contents in seed oil of *O. violaceus* (Fig. 6d).

## Discussion

Dissecting the metabolic and regulatory basis of the unique diOH-FA in *O. violaceus*, the potential industrial oil crop, is not only important for the improvement of seed oil quality but also can provide molecular resources for subsequent breeding endeavors [75]. Integrative analysis of metabolome and transcriptome is a high-effective approach for dissecting the regulatory mechanisms associated with key trait in numerous crops and fruits [43–45, 68, 76, 77]. In a previous study, it was discovered that di-OH FA is produced after 32 DAF in *O. violaceus* [29]. Therefore, we collected seeds from four different developmental times (21 DAF to 63 DAF) to conduct metabolome and transcriptome analyses to explore the dynamics of diOH-FA biosynthesis-related pathways (Fig. 1, Fig. 2).

A total of 1,003 metabolites and 22,479 expressed genes were detected in at least one developing stage of seeds. Based on the k-means cluster method, we further divided metabolites and expressed genes into three main clusters (Fig. 3). Flavonoids were mostly found in cluster II and cluster III which represented the accumulation period of the early or middle stage of seeds development, while the lipids preferred to presented in cluster I and cluster III, representing the early and the late stage of seeds development. We also found that free fatty acids mostly accumulated in the mature seed, especially for the key fatty acids, such as stearic acid, linoleic acid that play important roles in seed oil quality as reported in other oilseed crops [78–80]. The accumulation pattern we observed here is highly consistent with previous comprehensive studies in *B. napus*, implying the potential use of the wild species *O. violaceus* for enhancing seed quality in other related oilseed crops [69]. Taken together, our dataset could provide a valuable resource for the comprehensive study of metabolism regulation during seeds development of *O. violaceus*.

The productivity of diOH-FA in seeds determine the industrial quality of *O. violaceus*. In this study we mainly focused on four main biosynthesis process impacting the diOH-FA productivity, including fatty acid synthesis that provides precursor for diOH-FA synthesis, flavonoid synthesis that were known as negatively regulator to the seed oil content in *B. napus*, diOH-FA synthesis that directly influenced the diOH-FA content and TAG synthesis that were found affecting the storage of diOH-FA (Fig. 4). We performed a systematical gene identification method to characterize the genes related to the four main pathways using the gene annotation datasets of 19 available species in the family Brassicaceae [49]. Genes involved in flavonoid synthesis were mainly expressed in the early stage and most of them were enriched in cluster 3, which is consistent with the results reported in *B. napus* [69]. Meanwhile, most genes involved in fatty acid and TAG synthesis also had higher expression level in the early stage and enriched in cluster 3. Interestingly, we found that cluster2 contained all the structural genes directly involved in *O. violaceus*-specific discontinuous elongation pathway, including *FAD2, FAE1, KCR, HACD, ECR* together with *DGAT* genes, which might potentially regulate the storage of diOH-FA. We also found that the majority of structural genes in diOH-FA pathway remained two WGD copies (Fig. 4), implying the functional importance of WGD events in diOH-FA production. Consistent with previous analysis [13, 29], these WGD genes had similar expression pattern based on the heatmap analysis (Fig. 4, Fig. 6a), mainly expressed from 35 to 49 DAF, implying the potential gene dosage compensation effect in accumulating seed oil [81, 82]. As multiple gene copies produced by WGD provide enormous genetic diversity and some new compounds were born through the neofunctionalization of WGD gene pairs [2], WGD has been repeatedly demonstrated to contribute to evolutionary innovation for species adapting to changing environments and also holds promise for advancements in plant breeding [6, 9, 11, 83, 84].

To further identify transcription factors that might regulate the diOH-FA biosynthesis in seeds of *O. violaceus*, we further constructed TF-SG regulatory network. We found bHLH and bZIP TF families have the highest members showing high correlations to the structural genes of flavonoids biosynthesis. We found *TT2* from MYB family and *TT8* from bHLH family also showed high correlation to the flavonoid pathway. The results are in accordance with previous study using *A. thaliana* and *B. napus* as experimental materials, which also showed *TT2* and *TT8* could positively regulate the flavonoids content, changing the seed color from black to yellow and simultaneously increasing the fatty acid content of seed oil [34, 35, 85, 86]. Taken together, *TT2* and *TT8* of *O. violaceus* might play an important role in regulating flavonoids biosynthesis and future breeding efforts could focus on these transcription factors with the goal of improving the seed oil quality. In the fatty acid biosynthesis pathway, we also found several TF families such as bHLH, MYB, B3 have potential ability to increase the fatty acid content. CHS/TT4 competitively uses the common substrate maly-coA which is the upstream substrate of fatty acids biosynthesis to promote the flavonoids biosynthesis leading to the decrease of fatty acid contents. SAD catalyzed the first desaturation step to produce oleic acid which is the upstream substrate of diOH-FA. We assumed that changing the expression level of these two genes could indirectly change the diOH-FA contents. Based on the correlation analysis, we identified several TF candidates which could potentially regulated the two key genes, *SAD* and *CHS*/tt4 (Fig. 5C, D). These TF candidates could improve the seed oil quality of *O. violaceus* and more future molecular experiments are needed to validate their regulatory roles.

We next focused on the evolutionary history and regulatory relationship of diOH-FA biosynthesis pathway which directly determine the diOH-FA contents. Some key genes for diOH-FA synthesis have undergone functional divergence, for example the mutation for *FAD2* genes have changed its original function from desaturation to hydroxylase. Tracing the evolutionary history of *FAE1* genes indicate that the ancestor of Brassicaceae had two *FAE1* copies. Different species undergone asymmetrical retention of *FAE1* copies in different clades and the *FAE1* genes in clade-II might have the potential to produce diOH-FA. Using our identification method, we found that there are three *DGAT* genes in *O. violaceus* genome and their expression pattern were more diverged than other genes involved in diOH-FA synthesis (Fig. 4). It is important for further study to determine whether a specific copy acquired new function and specifically regulate the diOH-FA storage. Combining the mRNA-seq samples of different mature tissues, we found that MYB, B3 BH2H TF families play a important regulatory role in the diOH-FA synthesis pathway. In general, our study provides genetic basis of the regulatory pathways associated with the diOH-FA biosynthesis and pave the way for downstream breeding effort of this valuable industrial seed oil plants. Although our multi-omics networks have revealed multiple TFs that might regulate the expression level of diOH-FA related genes, further in vitro experiments such as LUC, EMSA, yeast one-hybrid assay and in vivo experiments such as Crisper-Cas9, Virus-Induced Gene Silencing (VIGS) are needed to verify the regulation mechanisms in order to precisely improve the seed oil quality of *O. violaceus*.

## Conclusions

In this study, we performed transcriptome and metabolome analysis to dissect the regulatory networks of diOH-FA related pathway from four different seed developing stages (21 DAF to 63 DAF) of *O. violaceus.* We divided all 1,103 annotated and 22,479 expressed genes of seeds into three main clusters based on their accumulation or expression patterns. The structural genes of fatty acid and flavonoid biosynthesis are highly active in the early seed developing stage of *O. violaceus*. Conversely, the structural genes of diOH-FA biosynthesis and *DGAT* genes are more active in the following stages. Through the correlation analysis between structural genes and TFs, we also identified several key transcription factors which potentially directly or indirectly regulate the diOH-FA biosynthesis, including *SAD, CHS/TT4, FAD2, FAE1* genes. We also trace the evolutionary history of diOH-FA related structural genes and find the majority of them still retain two WGD copies, and therefore, future studies are highly needed to dissect the role of WGD in driving formation of new traits in this potential industrial oilseed crop. Taken together, our findings provide new insights into the regulation of diOH-FA biosynthesis in *O. violaceus* and lay the foundation for future molecular validation and breeding efforts.

### Supplementary Information

**Additional file 1:** **Fig. S1.** K-means based cluster for (a) genes expression and (b) metabolites. **Fig. S2. **Phylogenetic tree of KCS family.

**Additional file 2: Tables S1-S8.**

## Data Availability

Transcriptomic data of different tissues of *Orychophragmus violaceus* have been deposited to China National Genomics Data Center (https://ngdc.cncb.ac.cn/) under accession ID (CRA012201).
