# Supplementary material for: Intergrative metabolomic and transcriptomic analyses reveal the potential regulatory mechanism of unique dihydroxy fatty acid biosynthesis in the seeds of an industrial oilseed crop Orychophragmus violaceus
Source: BMC Genomics. 2024 Jan 3;25:29. doi: 10.1186/s12864-023-09906-0 (PMC10765717; doi:10.1186/s12864-023-09906-0)
Supplement: Supplementary file 1 — Additional file 1: Fig. S1. K-means based cluster for (a) genes expression and (b) metabolites. Fig. S2. Phylogenetic tree of KCS family. [file 12864_2023_9906_MOESM1_ESM.docx]

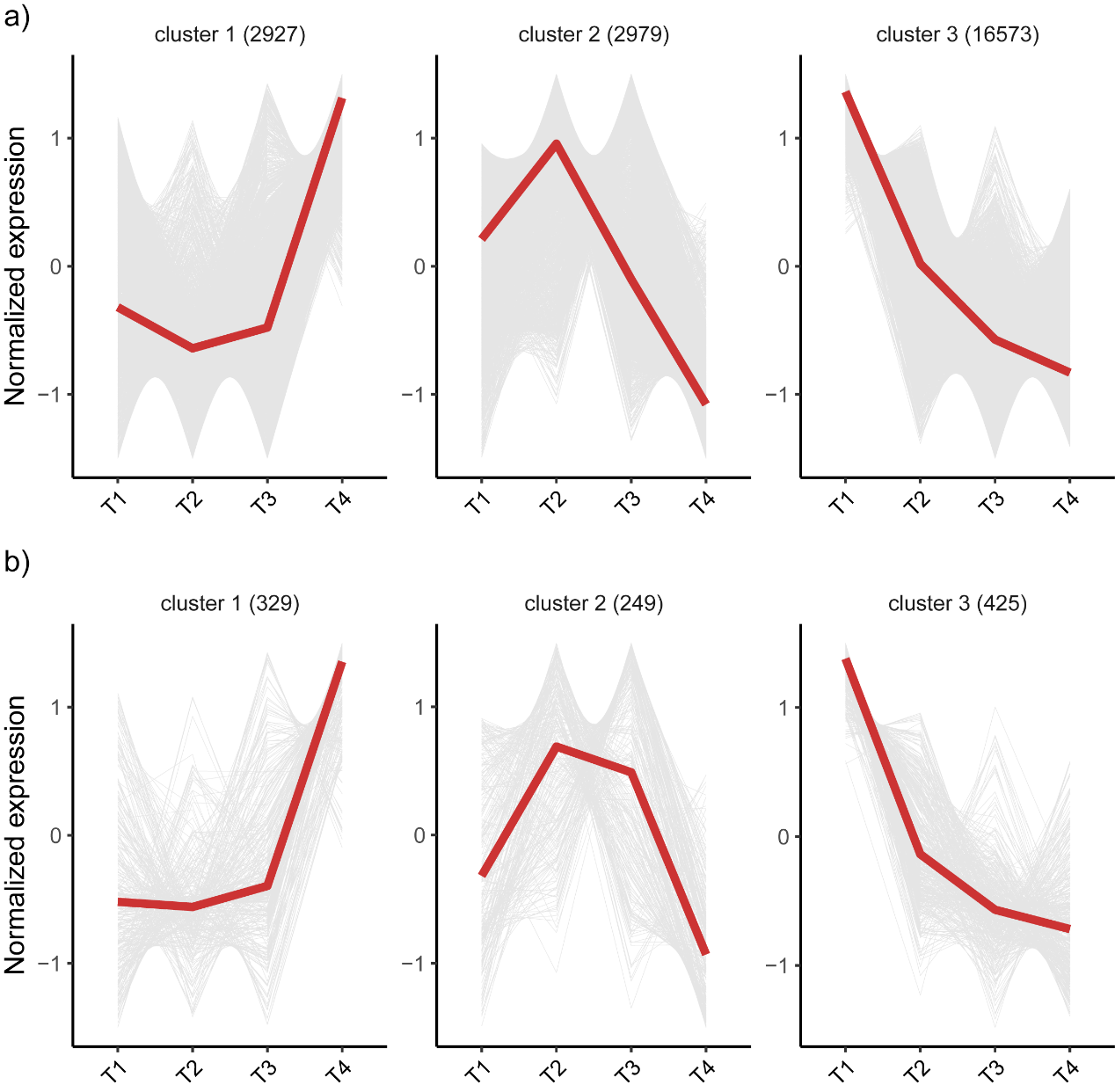


**Fig. S1.** K-means based cluster for (a) genes expression and (b) metabolites


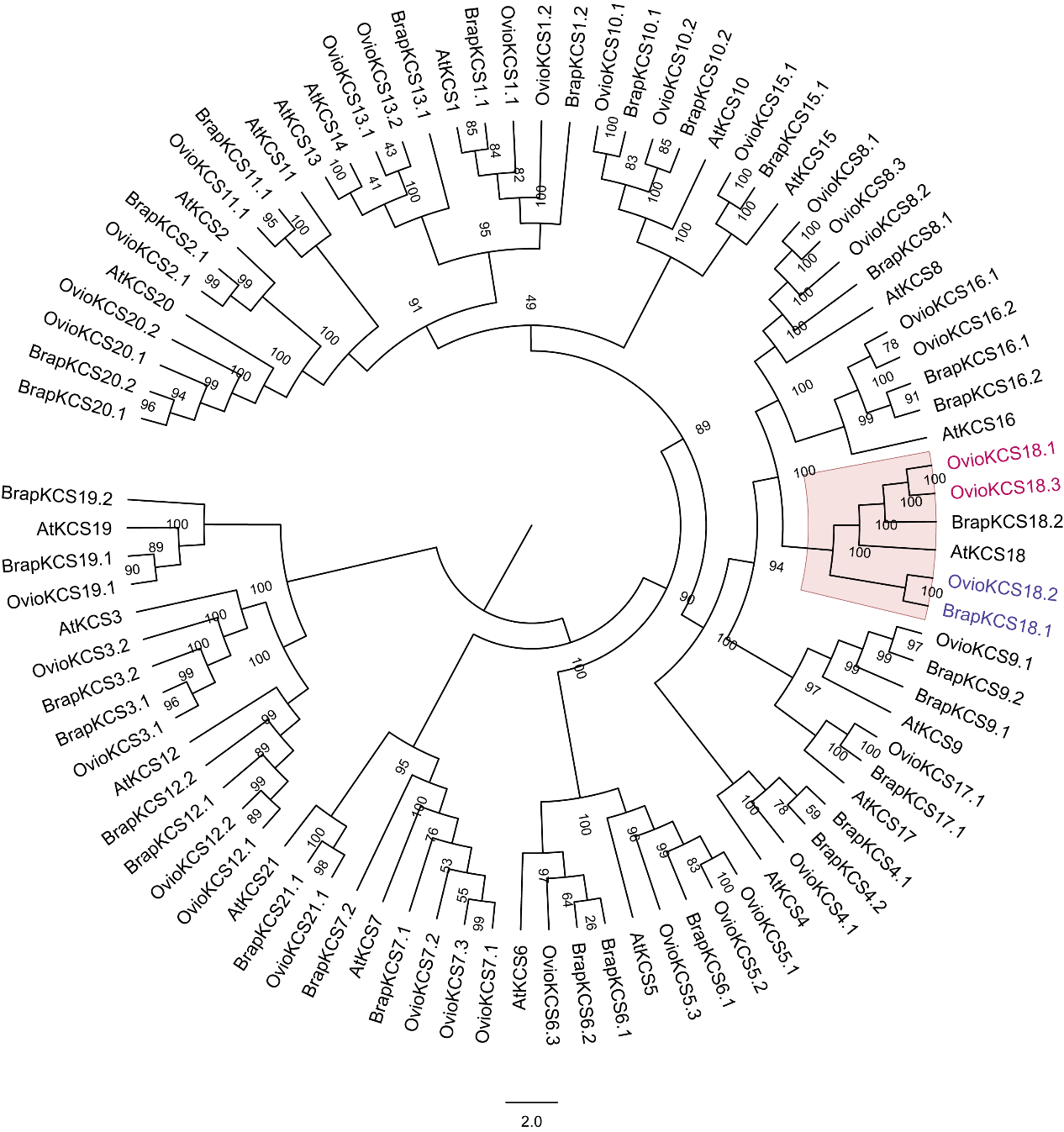


**Fig. S2.** Phylogenetic tree of KCS family.
